# Supplementary material for: Computational Pipeline for Targeted Integration and Variable Payload Expression in Bacteriophage Engineering
Source: ACS Synth Biol. 2025 Sep 22;14(10):4037–46. doi: 10.1021/acssynbio.5c00450 (PMC12538581; doi:10.1021/acssynbio.5c00450)
Supplement: Supplementary file 1 [file sb5c00450_si_001.pdf]

Supporting Information for: Computational Pipeline for Targeted Integration and Variable Payload Expression for Bacteriophage Engineering

Jonas Fernbach[1,2], Emese Hegedis[1], Martin J. Loessner[1], and Samuel Kilcher[1,*]

[1]Institute of Food Nutrition and Health, ETH Zurich, 8092 Switzerland
[2]Centre for ExoLife Science, University of Copenhagen, 2200 Copenhagen, Denmark
[*]E-mail: samuel.kilcher@hest.ethz.ch

Supporting Information

Supplementary Table S1: Primers used for amplification of the plasmid backbone and synthetic DNA strings.

**Primer Name**	**Sequence (5' → 3')**
Backbone Forward Primer	GTCGATGTTAAACCGTGTGCTCTACG
Backbone Reverse Primer	CGCGCTATTAATCGCAACATCAAACC
String Forward Primer	GGGGCTTTTATTTTGGTTTGATGTTG
String Reverse Primer	TTATAGTTTTGGTCGTAGAGCACACG

Supplementary Table S2: Synthetic DNA strings (pEDIT Phage K *nluc* donor templates) used to generate recombinant phages in this study.

**Synthetic String Name**	**Sequence (5' → 3')**
pEDIT Phage K::nluc_early	AAAGTCGAAGGGGGCTTTTATTTTGGTTTGATGTTGCGATTAATAGCGCGAGA<br>AGGTTATAATAAAGAATACTTGTCTAAAGCACTCAGATTAATCAATGACCATG<br>CTCCTAGGGAGTTAAGTTATGATTTTAATAATGTAGAAGCGGATGTTAATATT<br>CACACAATGTTATATGTTAAACCTGAAGATAGATTTATATATAAGGATATATC<br>CTATGACTTCCCGGGTGATTTAATTATTTGTATAGTTGATGATGATGCTATTG<br>TATACCATCAAGGTGAGCAGATTTCAGGTATTAGTATTTTAAGAATACTAGAA<br>GAGATATTTTAAGAGGAGGTAAATATATATGGTATTCACTTTAGAAGATTTCG<br>TAGGTGATTGGCGTCAAACTGCTGGTTACAACTTAGATCAAGTATTAGAACAA<br>GGTGGTGTATCATCATTATTCCAAAACTTAGGTGTATCAGTAACTCCAATCCA<br>ACGTATCGTATTATCAGGTGAAAACGGTTTAAAAATCGATATCCACGTAATCA<br>TCCCATACGAAGGTTTATCAGGTGATCAAATGGGTCAAATCGAAAAAATCTTC<br>AAAGTAGTATACCCAGTAGATGATCACCACTTCAAAGTAATCTTACACTACGG<br>TACTTTAGTAATCGATGGTGTAACTCCAAACATGATCGATTACTTCGGTCGTC<br>CATACGAAGGTATCGCTGTATTCGATGGTAAAAAAATCACTGTAACTGGTACT<br>TTATGGAACGGTAACAAAATCATCGATGAACGTTTAATCAACCCAGATGGTTC<br>ATTATTATTCCGTGTAACTATCAACGGTGTAACTGGTTGGCGTTTATGTGAAC<br>GTATCTTAGCTTAACGAGGATAAGTAATCATGATAGGAATAACAATATTAATT<br>ACGATAATGAGTATATCAACTATCTCTATGTATATTTATTTTTTAGTAGACTT<br>GATTCAGTCAATCAGATATAATAGTTTTGATAAGGTAATTAACGTCATAACAT<br>TTGTACTTATGACAGTTATAATAGCATCAGGTATTTTAGCTATACTTGGAATA<br>TAGAGCTCATTTAAGAAGCGGTTAAGTAGTTAGAGGGGATTTGTCCTAAAATA<br>GTATACCGCTTCTATATGGAAGGCTGAGAGGTCTTAGAATTGAAAGGAGAGAT<br>ATAATGATTCATATATTTTTAACTGATAGTTATGATAATAAAGTTTTAAATAC<br>TGTACTCAGATATATTAATACTACTAGTGATAGAGAGCTTAGTAGTCGATGTT<br>AAACCGTGTGCTCTACGACCAAAACTATAAAACCTTTAAG

Synthetic String Name	Sequence (5’ → 3’)
pEDIT Phage K::nluc_mid	AAAGTCGAAGGGGGCTTTTATTTTGGTTTGATGTTGCGATTAATAGCGCGGCA<br>ACTTTCTCCATTCGAACAATCAAAACATCACACACAAACAATTTATGATGATA<br>TTCAAGTACTAGATATGATTATTTCTAAAGGTGCAAAAGGATTAGAGTTTGTG<br>GAAACTTTAGACCCTGCTTTAATGATACGTGCAATGGAAACTAAAGATAAGAT<br>TACCGGAAATCAATTAAAAGGTATGTCATTTATTGGACTTAGAGAATTACAAT<br>TAAAACAAACAGCTCAAGATACAGCTATGAGTGAAGTATTATTAGAATTTATA<br>CCTGAAGAGAAACATGAAGAGGTATTACAACGATTAGAAGAACTACAAAATGA<br>ATTCTACAAAAATCTAGATTTAGATGAAGAAAGTAGAAAATTAAAAGAAGCTC<br>TTGATAGAGTAGGCTATACAATTTAGGAGGAGGTAAATATATATGGTATTCAC<br>TTTAGAAGATTTCGTAGGTGATTGGCGTCAAACTGCTGGTTACAACTTAGATC<br>AAGTATTAGAACAAGGTGGTGTATCATCATTATTCCAAAACTTAGGTGTATCA<br>GTAACTCCAATCCAACGTATCGTATTATCAGGTGAAAACGGTTTAAAAATCGA<br>TATCCACGTAATCATCCCATACGAAGGTTTATCAGGTGATCAAATGGGTCAAA<br>TCGAAAAAATCTTCAAAGTAGTATACCCAGTAGATGATCACCACTTCAAAGTA<br>ATCTTACACTACGGTACTTTAGTAATCGATGGTGTAACTCCAAACATGATCGA<br>TTACTTCGGTCGTCCATACGAAGGTATCGCTGTATTCGATGGTAAAAAAATCA<br>CTGTAACTGGTACTTTATGGAACGGTAACAAAATCATCGATGAACGTTTAATC<br>AACCCAGATGGTTCATTATTATTCCGTGTAACTATCAACGGTGTAACTGGTTG<br>GCGTTTATGTGAACGTATCTTAGCTTAAATAGTGACGTTAGAGTAATGGCAGA<br>TGAGATTAGTTTAAATCCAATACAAGATGCTAAGCCAATTGACGATATAGTAG<br>ATATCATGACATACTTAAAAAACGGGAAAGTACTGAGAGTTAAACAAGACAAC<br>CAAGGAGATATCCTTGTTAGAATGAGTCCAGGGAAACACAAATTTACTGAAGT<br>ATCTAGAGACTTAGATAAAGAATCATTCTACTATAAAAGGCATTGGGTTCTCT<br>ATAATGTATCTGTTAACTCTCTTATAACATTTGATGTTTATCTAGATGAAGAA<br>TATTCAGAAACAACTAAGGTTAAGTATCCTAAAGATACTATTGTAGAATATAC<br>AAGAGAAGACCAAGAAAAAGATGTTGCTATGATTAAAGAAATACTTACAGATA<br>ATAAAGTCGATGTTAAACCGTGTGCTCTACGACCAAAACTATAAAACCTTTAA<br>G

**Synthetic String Name**	**Sequence (5' → 3')**
pEDIT Phage K::nluc_late	AAAGTCGAAGGGGGCTTTTATTTTGGTTTGATGTTGCGATTAATAGCGCGTAT<br>TGATAAATAAGTCAACAGTTTTCTAAAAATAATTTAAATTATTTTGAAGAATA<br>CTTTAATATCAAGGGTTACAAGAGAAAAAGTACGTATTTAGAAAATAAGGAGT<br>ACTCCTATTATATATAATTATATTCTGATATAGAGTAATAAATAATATTAAAT<br>ATATAATTATAATTAATAAGGTTGGGAAAATTGATATAAACATAACTGATACT<br>GCTTATAGATACTCAGTATAAAAGTAAAATCCCTTAGTATCAGTACTTACAGG<br>CAAAAAAGTACGTATTTAGAAAATAAGGAGCTCTGCTATTATAGTTATATATA<br>TTTATTACTATTATTAATTACTATTTAAATATATAATTATAATTAACAATGTT<br>AGAAAGTCAACAATAGTATAAATAAAGAGGAGGTAAATATATATGGTATTCAC<br>TTTAGAAGATTTCGTAGGTGATTGGCGTCAAACTGCTGGTTACAACTTAGATC<br>AAGTATTAGAACAAGGTGGTGTATCATCATTATTCCAAAACTTAGGTGTATCA<br>GTAACTCCAATCCAACGTATCGTATTATCAGGTGAAAACGGTTTAAAAATCGA<br>TATCCACGTAATCATCCCATACGAAGGTTTATCAGGTGATCAAATGGGTCAAA<br>TCGAAAAAATCTTCAAAGTAGTATACCCAGTAGATGATCACCACTTCAAAGTA<br>ATCTTACACTACGGTACTTTAGTAATCGATGGTGTAACTCCAAACATGATCGA<br>TTACTTCGGTCGTCCATACGAAGGTATCGCTGTATTCGATGGTAAAAAAATCA<br>CTGTAACTGGTACTTTATGGAACGGTAACAAAATCATCGATGAACGTTTAATC<br>AACCCAGATGGTTCATTATTATTCCGTGTAACTATCAACGGTGTAACTGGTTG<br>GCGTTTATGTGAACGTATCTTAGCTTAAAAAGTGACTACTTAAAGTCACTCAA<br>TAATTAGAATACTATTTTAAAAGATTCTATTCTGTTTGGATTAATATATACTT<br>GAGGTGAAGTTATAGCACTTTCAGTATATACTTTTATAGAGGTTTCATCCATT<br>CCTCTTAACATATAATCTATATCTTGCCTATTGTAACTCTTTTCATCAGTAGA<br>TACTAAAAAGTATTTAGCTCCACTTGACATTGTTATTTCAATATGTTTTGACA<br>TCTACAATCTCTGCTATGCAAATTTGTTAAAGACAAAGGATAATATAGCTCCT<br>AGAACAAGTAAAAGAACCTTCTCAGTTGTATCCTTTTTCTTAGTATCCTTAGT<br>TTTTGTACTTTCAGCAAGTTCTGAAATCTTTTCATCAAGTCTTTCTAATTGGA<br>CGTAAGTCGATGTTAAACCGTGTGCTCTACGACCAAAACTATAAAACCTTTAA<br>G
